# Supplementary material for: Predicting visual function by interpreting a neuronal wiring diagram
Source: Nature. 2024 Oct 2;634(8032):113–23. doi: 10.1038/s41586-024-07953-5 (PMC11446822; doi:10.1038/s41586-024-07953-5)
Supplement: Supplementary file 6 — CRF and ERF predictions for individual TmY4 and TmY9 cells. Analogous to Supplementary Data 3, but for TmY target types. Shown are the top four monosynaptic pathways, the strongest pathway passing through each of the top ten intermediary types (ranking from Extended Data Fig. 7), and the trisynaptic pathway Tm1–TmY–Dm3–TmY (see the section entitled Prediction of spatial normalization). [file 41586_2024_7953_MOESM6_ESM.zip › DataS4/TmY9q/720575940619148869.pdf]

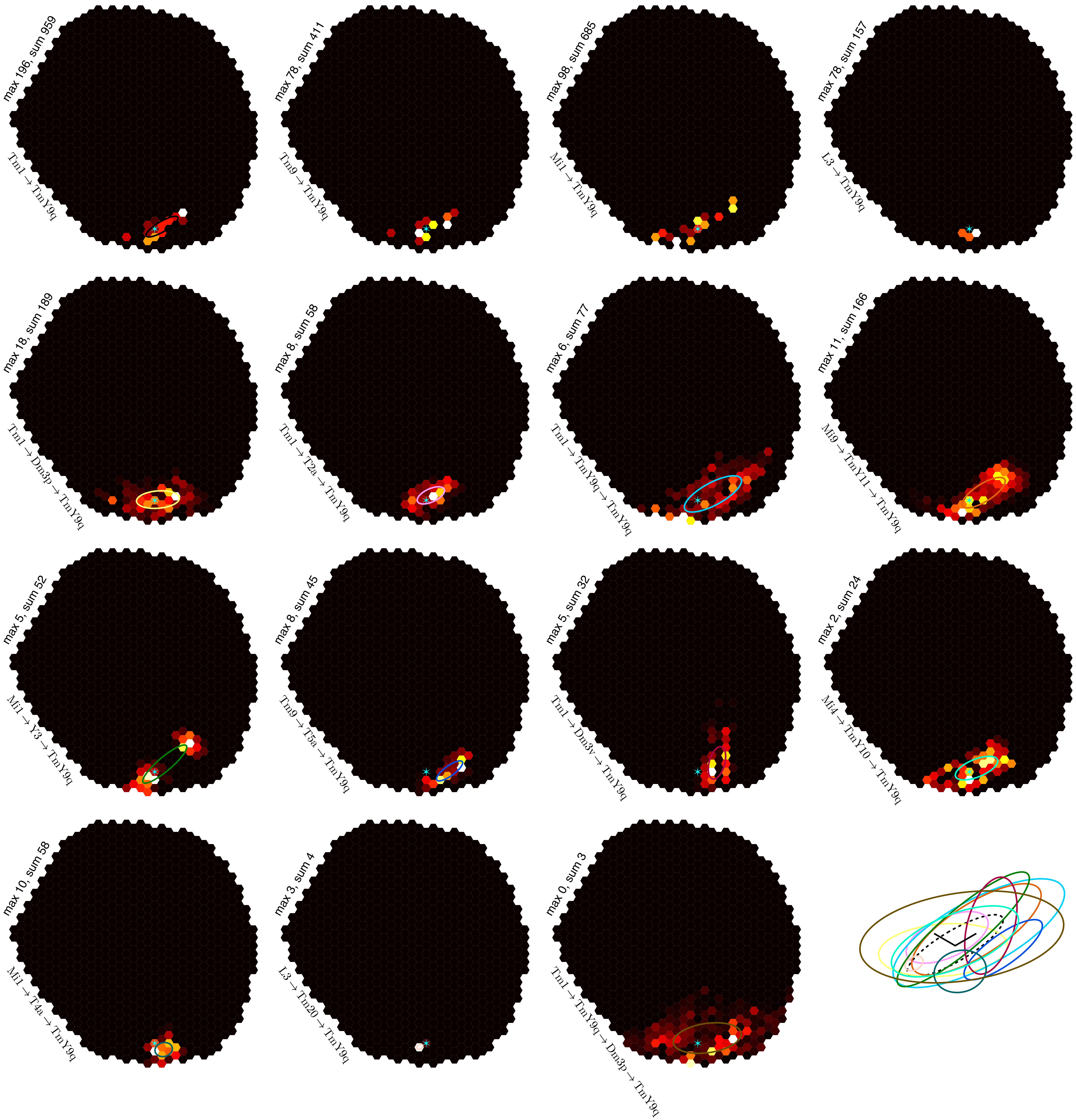
max 196, sum 959
Tm1 → TmY9q
max 78, sum 411
Tm9 → TmY9q
max 98, sum 685
Mi1 → TmY9q
max 78, sum 157
L3 → TmY9q
max 18, sum 189
Tm1 → Dm3p → TmY9q
max 8, sum 58
Tm1 → T2a → TmY9q
max 6, sum 77
Tm1 → TmY9q → TmY9q
max 11, sum 166
Mi9 → TmY11 → TmY9q
max 5, sum 52
Mi1 → Y3 → TmY9q
max 8, sum 45
Tm9 → T5a → TmY9q
max 5, sum 32
Tm1 → Dm3v → TmY9q
max 2, sum 24
Mi4 → TmY10 → TmY9q
max 10, sum 58
Mi1 → T4a → TmY9q
max 3, sum 4
L3 → Tm20 → TmY9q
max 0, sum 3
Tm1 → TmY9q → Dm3p → TmY9q
